# Sperm, Neutrophil and Vascular Alterations in Advanced Paternal Age Model and the Nutraceutical Effect of Açaí to Mitigate Health Vulnerability in the Male Offspring

**DOI:** 10.3390/biology15131086

**Published:** 2026-07-06

**Authors:** Amanda Guimarães de Araujo, Eder Henrique Alves Pinto, João Carlos Araújo de Oliveira, Beatriz Guerra Pompermayer, Stephany de Souza, Valéria dos Santos, Mônica Marques Telles, Vanessa Vendramini

**Affiliations:** 1 Laboratory of Reproductive and Developmental Biology (LaBReD), Department of Morphology and Genetics, Paulista School of Medicine, Federal University of Sao Paulo-EPM/UNIFESP, Sao Paulo 04023-900, Brazil; guimaraes.amanda@unifesp.br (A.G.d.A.);; 2 Department of Physiology, Discipline of Nutrition, Paulista School of Medicine, Federal University of Sao Paulo-EPM/UNIFESP, Sao Paulo 04021-001, Brazil

**Keywords:** inheritance, anthocyanin, intergenerational, inflammaging, systemic, motility, NLR, systolic

## Abstract

Açaí is considered a super fruit for its high concentration of flavonoid polyphenols, predominantly anthocyanins. The nutraceutical effect of açaí as an anti-inflammatory and antiapoptotic agent has been reported. This study aimed to investigate whether açaí protects paternal contribution against accelerated aging using the D-galactose model. Wistar rats (*n* = 24) were treated by voluntary consumption with vehicle paste (C) or a mixture with 200 mg/kg of D-galactose (DG) or 300 mg/kg of lyophilized açaí (A); D-galactose and açaí (DGA) were given separately. Fathers (F0) and offspring (F1) underwent Doppler ultrasound (aorta and kidney) and spermatic and immune cell analyses. In the F0 generation, the DG group showed altered epididymis weight, sperm quality, aortic diameter, neutrophil-to-lymphocyte ratio, systolic velocity and renal resistivity index. Açaí positively modulated sperm, neutrophil and vascular alterations in F0. The male F1 of the DG group showed lower body mass, as well as a greater number of abnormal spermatozoa and leukocyte DNA breaks; however, the DGA group had a significant increase in body mass and the NRL was positively correlated with leukocyte DNA breaks. Our data indicate that age acceleration, induced by D-galactose for 30 days, causes innate physiological dysfunction and compromises sperm contribution, which was positively modulated by açaí. The preconception use of lyophilized açaí also has some positive impact on intergenerational health, but the topic still deserves further investigation.

## Simple Summary

Fatherhood after forty can increase the risk of developmental challenges and health complications for children. This study aimed to investigate whether açaí, a fruit rich in antioxidants, protects the male parent and the first generation against the harmful effects of aging. Using the rat model to simulate the aging process through treatment with excessive amounts of a simple sugar, we showed the significant health benefits provided by açaí in the fathers, improving sperm parameters, circulation and systemic inflammation. Interestingly, a few indicators of health vulnerabilities were altered in the male pups, which were partially controlled by açaí. In conclusion, açaí successfully improved fathers’ health but had a modest positive impact on the male rat descendants. Our findings suggest that the use of anthocyanin-rich foods, such as açaí, in preconception may be a promising strategy to protect men’s general health and semen quality, potentially engendering the transmission of advantageous features to the general health of the first generation. On the other hand, the effects of daily açaí consumption passed on from fathers to children can have other implications that were not described in this study and should be better investigated.

## 1. Introduction

We are currently witnessing a global shift in the paradigm of the fertility window, with an increasing number of couples choosing to start families after the age of 35 [1]. Although late parenthood is not a new phenomenon, the rising trend in births among older fathers is accompanied by a growing body of evidence regarding health issues in their descendants. This has prompted a critical debate on the necessity of developing new public policies for preconception care [2].

Aging triggers time-dependent changes that disrupt proper physiological processes, presenting a significant challenge for healthcare systems [3]. The link between organic aging and low-grade systemic inflammation—termed “inflammaging” by Franceschi [4]—establishes a cycle of functional decline driven by immunosenescence [5].

The interconnection between oxidative stress, inflammation, and senescence, when applied to the male reproductive system, creates a conductive scenario for the functional decline associated with advanced paternal age (APA), or fatherhood after the age of 40. APA is currently considered an important risk for pregnancy loss up to 20 weeks of gestation [6], as well as low birth weight [7,8]; in addition, it has been related to the escalating incidence of autism spectrum disorders [9,10,11].

Paternal inheritance is transmitted to the offspring through genetic and epigenetic information delivered by either the nuclear chromosomes or complementary and indispensable messenger RNA acquired by the sperm in the testis, during spermiogenesis, or in the epididymis, through special packaged epididymosomes [12]. Therefore, an aged sperm may carry diverse misinformation from multiple origins, causing disruptive morphogenesis, organ malfunction, and cancer development. Undoubtedly, neurodevelopmental and psychiatric disorders are significantly elevated in fatherhood after 40–50 years [11,13,14,15]. Moreover, studies dedicated to the subject have already linked APA to a higher risk of developing cardiovascular [16] and hematological diseases [17] in the offspring, which can differ in a sex-specific manner.

Among the hallmarks of aging, genomic instability [18,19], reduction in DNA methylation [20], telomere shortening [21], loss of proteostasis [22], dysregulation in nutrient detection [23] and mitochondrial dysfunction [24] are targets that stand out. In this context, metabolic dysfunction also represents a sensitive point that invariably reverberates throughout the entire body, with repercussions on the reproductive system [23,24,25].

Testicular aging impairs spermatogenesis and sperm quality, key indicators of male reproductive health [26]. The age-related decline in intrinsic antioxidant capacity leads to a progressive increase in free radical damage, affecting both the germline and somatic cells [27,28]. Notably, this includes the endothelial cells [29], which are essential for the nourishment of the seminiferous epithelium [30]. Not surprisingly, the male reproductive system of an advanced age man is frequently associated with conditions such as hypogonadism, prostatic hypertrophy, and erectile dysfunction [26,31].

In middle-aged men, some seminal parameters, especially motility, morphology and the level of DNA fragmentation, are age-sensitive parameters [26,32] and have been related to other systemic indicators for detecting comorbidities, such as metabolic alterations, systemic inflammation, or health risks linked to environmental factors [26,33,34].

Systemic biological indicators screen for infertility-related comorbidities [34,35], providing diagnostic and therapeutic decision usefulness. These include classic indicators of oxidative stress, total antioxidant capacity, as well as specific inflammatory markers. For instance, the gradual increase in the Neutrophil-to-Lymphocyte Ratio (NLR) has been consistently correlated with inflammaging and immunosenescence [4,36].

Despite the evidence of structural changes in DNA as a hallmark of aging [19], along with DNA methylation modifications [37] and insufficient microRNA content [38]—all of which constitute plausible mechanisms for the transgenerational transmission of metabolic traits and other health issues—these targets are not yet considered in the context of intergenerational risk diagnosis and preconception counseling [39]. The identification of some biological targets is not easily accessible, due to the requirement for specialized technical training and/or procedures involving higher costs. However, since aging is not a local event, the factors that increase the risk of paternal inheritance and APA might also be indicated by systemic aging marks.

D-galactose is a reducing monosaccharide present in milk and dairy products, not harmful when consumed with parsimony. However, in excess, this reducing sugar is highly reactive with free amines in proteins, thus forming advanced glycation end products [40,41,42]. The age acceleration induced by D-galactose has been largely explored to mimic natural aging because it exhibits phenotypes such as neurological impairment and decreased immune responses, as a consequence of reduced antioxidant enzyme activity and increased ROS production. These alterations lead to mitochondrial dysfunction, inflammation and organ failure, replicating natural aging and providing a convenient platform for studying therapeutic interventions [42,43,44].

Açaí (*Euterpe oleracea* Mart.) effectively controls the oxidative stress associated with various metabolic alterations, owing to its antioxidant and anti-inflammatory potential [45,46,47]. The fruit is particularly rich in flavonoid polyphenols, particularly cyanidin-3-glucoside [47], a natural anthocyanin. Açaí extracts have been successfully applied in the management of cardiovascular disorders, obesity, neurodegenerative conditions, liver diseases, and neoplasms, stress tolerance, and longevity [33,46,48,49].

Therefore, this study investigated the systemic and intergenerational nutraceutical effects of freeze-dried lyophilized açaí, following a 30-day induction of accelerated aging by D-galactose in male rats. Our central hypothesis is that açaí attenuates the oxidative and inflammatory status associated with accelerated senescence—assessed through sperm, immune cells, and circulatory parameters—and that the protective benefits are extendable to the offspring.

## 2. Methods

### 2.1. Approval from the Local Ethics Committee

The experimental protocols for this study were conducted in accordance with the practices recommended by the ARRIVE guidelines 2.0 and the National Council for the Control of Animal Experimentation (CONCEA/MCTI/Brazil). The proposed protocols were approved by the Ethics Committee on the Use of Animals of the Federal University of São Paulo (CEUA/UNIFESP, protocols: 9534050524).

### 2.2. Animals and Group Composition

Male Wistar rats (*Rattus norvegicus* alb) (*n* = 24) were obtained from the Center for the Development of Experimental Models for Medicine and Biology (CEDEME) of the Paulista School of Medicine (EPM), Federal University of São Paulo (UNIFESP). During the experiments, they were kept at the animal facility of the Laboratory of Neurosciences (UNIFESP/Brazil), respecting a 7-day adaptation period before the experiment started. They were housed in polypropylene cages, with up to three animals per unit, under an environment of wood shavings, controlled hygiene, a light/dark (7 a.m./7 p.m.) cycle and controlled temperature (23–25 °C). The rats were provided with commercial food (Nuvilab, Quimtia S/A, Curitiba, Paraná, Brazil), and water was provided ad libitum during the experiment. Environmental enrichment was also provided with sterilized materials, such as brown paper rolls and paper towels.

The experimental groups were randomly formed (*n* = 6 males/group) after a 3-day training period of handling before introducing the voluntary consumption protocol (vcp) placed in a watch glass, as an alternative stress-free treatment method of intragastric treatment [50]. A melon paste (*Cucumis melo*), at a dosage of 2 g/kg/day, was used as a vehicle facilitator to be mixed with the targeted compounds. After the training, the rats were offered treatments according to the experimental groups: Control, which consumed 2 g/kg of body weight of melon paste; Açaí (*Euterpe oleracea* Martius), which consumed 300 mg/kg of freeze-dried açaí (Açaí da Fábrica, Itu, Sao Paulo, Brazil; Production batch: 789864384501), mixed with melon paste, for 5 days/week (in the evening); D-galactose, which consumed 200 mg/kg of body weight of D-galactose (G0750-Sigma, São Paulo, Brazil) mixed with melon paste in daily doses (at noon); and D-galactose/Açaí, which consumed a mixture of melon paste with both 200 mg/kg D-galactose (at noon) and 300 mg/kg of freeze-dried açaí (in the evening). The treatment period for all groups was thirty days.

### 2.3. Obtaining the F1 Generation

At the end of treatments, three males per group were placed in cohabitation with untreated females in estrus (2 females/males; totaling *n* = 24 females) at 6:00 PM. The following morning, mating was confirmed by the presence of sperm in the vaginal smear. Pregnant females were monitored throughout the prenatal period. On the third postnatal day (3PND), pups were individually weighed and sexed through external anatomical inspection. Eight pups were kept with each lactating female until the end of the weaning period; to standardize litter size, priority was given to the male pups, and culling of supernumerary pups was applied. The female pups maintained to complete the litter size were euthanized on the day of weaning using the same euthanasia procedures described for the male offspring, which were monitored for anatomical/morphological anomalies and body mass gain twice a week up to the 70th postnatal day (70PD).

### 2.4. Organ and Cell Sampling

For sample collection, both paternal (F0) and the male first generation (F1) were anesthetized by intraperitoneal injection with a combination of ketamine (1 mL/kg) and xylazine (0.6 mL/kg). After the ultrasound analysis and organ and cell preparations, a humane endpoint was established through thoracic decompression. Testes, epididymis, liver, kidneys, blood (by cardiac puncture), and spermatozoa were sampled accordingly. The organs were weighed immediately after collection using a semi-analytical scale. Blood smears were prepared fresh on a slide for leukocyte counting.

The left epididymis was used for fresh sperm sampling, and the right epididymis was immediately frozen at −80 °C. Fresh spermatozoa were collected by isolating the caudal and head portions of the left epididymis, which were immersed in 4 mL of PBS (phosphate-buffered saline; pH = 7.4), and after perforations, extravasation of the efferent duct contents. The solution containing spermatozoa was filtered (100 microns; Merck Millipore™, São Paulo, Brazil) centrifuged, resuspended, and sampled [51]. Blood and sperm samples were stored at −80 °C.

### 2.5. Sperm Analysis

Sperm parameters (concentration, morphology and motility) were evaluated following WHO guidelines (2021), as well as the methodology proposed by Filler [52].

Concentration: The right epididymis was thawed, and its portions, (1) head + body and (2) tail, were homogenized and diluted (20 times); sperm contained in each portion were counted in the Neubauer chamber (5 internal quadrants; 4 chambers per animal). The resulting averages of the counted fields were expressed in millions/mL.

Motility: Fresh samples collected from the left epididymis were diluted (20 times) and analyzed under light microscopy (200× magnification). The number of progressively motile spermatozoa was counted.

Morphology: A sample was taken from the homogenate, and smears were obtained on slides, stained with the Papanicolaou method for analysis using light microscopy (50× objective). Sperm displaying abnormal external morphology (head, midpiece and cauda) were scrutinized [53].

### 2.6. Neutrophil-to-Lymphocyte Ratio (NLR)

Blood cells were collected on the day of euthanasia by cardiac puncture for the preparation of blood smears and leukocyte count. Lymphocytes and neutrophils were counted under light microscopy (50× objective) according to their morphological profile (100 cells/slide), and the neutrophil–lymphocyte ratio was then calculated [54]. This biomarker is largely used to measure the balance between the innate immune response (neutrophils) and adaptive immunity (lymphocytes) [55].

### 2.7. DNA Integrity

The comet assay was performed to evaluate the presence of DNA breaks in leukocytes, a technique based on the analysis of individual cells subjected to horizontal electrophoresis on agarose gel [56]. Slides were coated with 1% normal agarose diluted in 1 × PBS and air-dried. Cell samples (5 µL) were individually mixed in 115 µL low-melting-point agarose (at 0.5% diluted in 1 × PBS), previously heated and maintained at 37 °C. An amount of 100 µL of the mixture was placed on the pre-coated slides, covered with a coverslip and immediately transferred to refrigeration at 4 °C for 5–10 min to solidify the agarose. After removing the coverslips, the slides were immersed in chilled lysis solution (2.5 M NaCl, 100 mM EDTA, 10 mM TRIS, pH 10, supplemented with 1% Triton X-100 and 10% DMSO at the time of use) and kept refrigerated (4 °C) overnight. The following day, slides were briefly washed with distilled water and electrophoresis was conducted using a horizontal chamber containing a chilled solution of 1 × TBE (Tris/Borate/EDTA) buffer in sufficient volume for complete immersion of the slides. The entire procedure was performed under controlled low temperature (4 °C) and protected from light to avoid further damage to the DNA. Electrophoresis was performed for 20 min at a constant voltage of 25 V and under an average current of 300 mA. Subsequently, the slides were neutralized with a TRIS-HCl buffer (0.4 M, pH 7.5) and stored at 37 °C for 2 h. The analysis was undertaken by staining each slide with 50 µL of ethidium bromide (1.5 mL of TBE to 2 µL of dye) for 15 min in the dark. Individual cells counted 50 cells per slide (totaling 100 cells per animal) using a fluorescence microscope (Nikon Eclipse CI, Tokyo, Japan) equipped with a Nikon DS-QiMC camera, and the images were saved using the Lucia Comet Assay software v.7.02.0 (Laboratory Imaging s.r.o., Praha, Czech Republic) for subsequent classification of the cells according to their characteristics: intact, round and well-defined nucleus or with no damage (score 0); well-defined nucleus with some dispersion of DNA around it or with low damage (score 1); comet formation with the nucleus still evident and the presence of a tail (up to ⅔ of the size of the nucleus) or with moderate damage (score 2); comet formation with a tail greater than ⅔ of the size of the nucleus or severe damage (score 3) when the nucleus cannot be identified.

### 2.8. Ultrasound with Doppler

Rats under anesthesia had their abdominal hair removed using Depimiel depilatory cream (Depimiel do Brasil, Curitiba, Brazil). Subsequently, ultrasound gel was used in the ultrasonographic analysis of the kidneys and abdominal aorta, performed using the Visual Sonics Vevo 2100 equipment, located in the Animal Imaging Center (EPM/UNIFESP) under a controlled room temperature (37 °C). Renal parameters were obtained by using B-mode (brightness mode) at a frequency of 21 MHz, power 100%, 2D gain of 27 dB and PRF (pulse repetition frequency) of 2 kHz. Doppler images were acquired with a Doppler gain of 24 dB and PW (pulsed wave) measurements were performed by adjusting the parameters up to a value of 200 to obtain spectral curves suitable for hemodynamic analysis. For the evaluation of the abdominal aorta in B-mode, the same parameters were used, except for the 2D gain, which was adjusted to 29 dB. A Doppler gain of 28 dB was standardized and PW acquisitions were performed with parameters ranging from 400 to 600, adjusted according to the blood flow velocity in the evaluated vessel [57].

### 2.9. Statistical Analysis

The software GraphPad Prism (version 8.0.2) was used in all of the statistical analyses presented here. The data were first subjected to the Shapiro–Wilk test to check for normality distribution. One-way or two-way ANOVA (analysis of variance) tests were used when the data passed the normality test. When differences between groups were detected (*p* < 0.05), Tukey’s post hoc test was applied. Non-parametric data were analyzed using the ANOVA on ranks (Kruskal–Wallis) followed by Dunn’s post hoc test. Pearson’s correlation was also used to assess the strength and direction of the linear relationship between two continuous variables. Statistical differences with *p* < 0.05 were considered significant following multiple comparison tests. To provide data transparency, statistical tests are presented alongside the results.

## 3. Results

### 3.1. Sperm Motility and Morphology Were Reduced; Epididymis Weight Was Increased, but Açaí Modulated These Alterations in the Paternal Generation

Body mass and organ weights (absolute and relative) were not different among the groups, as presented in Table 1. However, the DG group presented higher absolute and relative epididymis weights compared to the control groups C and DGA but no difference compared to Group A (*p* = 0.08). Conversely, the DGA group did not show significant differences compared to C (*p* = 1.00) and A (*p* = 0.92) in these same parameters (Group C with *p* = 0.93 and Group A with *p* = 0.95).

**Figure 1:**
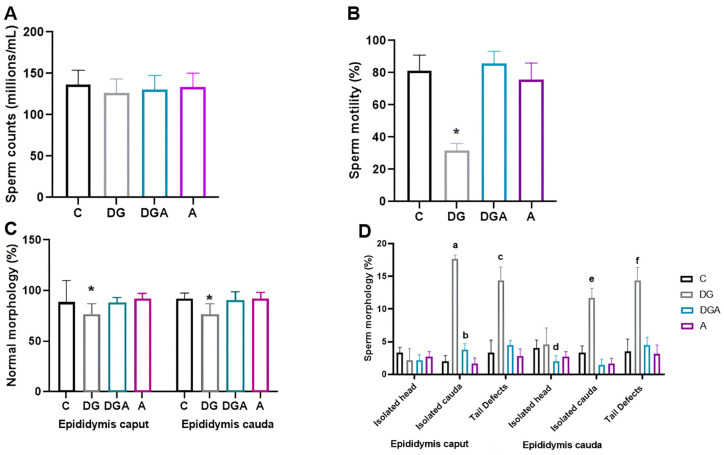
Sperm analyses of the paternal generation (F0). (**A**) Sperm concentration (millions/mL). No statistical significance was found (ANOVA one-way). (**B**) Sperm progressive motility. One-way ANOVA followed by the Tukey test. * DG > C, DGA and A. (**C**) Sperm classified with normal morphology collected from the epididymis caput and cauda. * DG > C, DGA, A. (**D**) Types of sperm morphological alterations in the epididymis caput and cauda. a: DG > C, DGA, A; b: DGA > A; c: DG > C, DGA and A; d: DGA < C and DG; e: DG > C, DGA, A; f: DG > C, DGA and A. Values expressed as mean ± SDV (*n* = 6/group).

Although the sperm counts did not differ among the groups (Figure 1), the percentage of motile sperm was significantly reduced in the DG group (*p* < 0.0001; Figure 1); alterations were also seen for normal morphology from both the epididymis head (*p* = 0.001; 0.002; <0.0001 respectively) and cauda (*p* < 0.0001; 0.0001; <0.001 respectively) after applying Tukey’s post-test (Figure 1C). Figure 1D categorizes the most relevant morphological alterations observed, which were significantly different between groups (two-way ANOVA; F = 10.89; *p* = 0.001). Sperm isolated tail, isolated cauda and tail defects in both the epididymis head and cauda were altered in the DG group (*p* < 0.0001), while the DGA group only showed an increase compared to Group A (*p* = 0.04). In the epididymis cauda, the number of isolated heads in the DGA group was lower when compared to groups C and DG (*p* = 0.03 and 0.005, respectively).

### 3.2. Peripheral White Blood Cells: Marks of Inflammaging in the Treated Males (F0)

**Figure 2:**
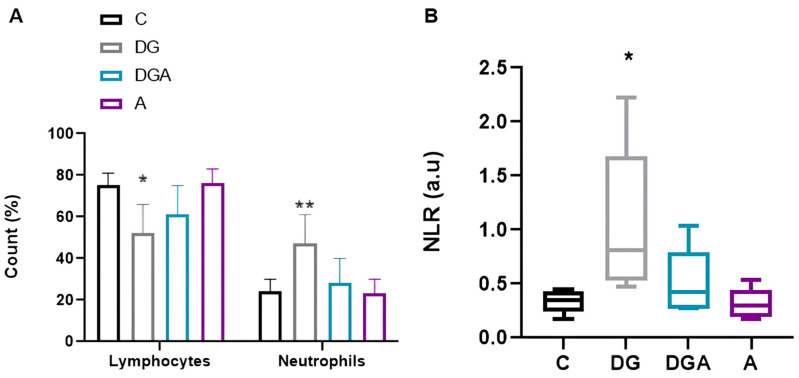
Quantification of lymphocytes and neutrophils in blood samples in the paternal generation (F0). (**A**) Absolute counts. Values expressed as mean ± SDV. (**B**) Neutrophil–lymphocyte ratio (NLR). Data expressed in median and interquartile intervals (*n* = 5/group). * DG < C and A; ** DG > C and A.

The neutrophil and lymphocyte counts and the neutrophil-to-lymphocyte ratio, a well-established mark of immune system imbalance, differed among the groups, as shown in Figure 2A (two-way ANOVA; F = 4.09; *p* = 0.05) and Figure 2B following Kruskal–Wallis (χ2 = 3.68, *p* = 0.02). The lymphocyte counts in the DG group (Figure 2A) was lower compared to the C and A groups (*p* = 0.01 for both comparisons) but not to the DGA group (*p* = 0.54); on the other hand, the number of neutrophils was significantly higher in the DG group compared to all the other groups (C: *p* = 0.01; A: *p* = 0.01; DGA: *p* = 0.04).

It is relevant to point out that the DGA group showed no significant difference compared to the C (*p* = 0.49) or A (*p* = 0.37) groups in the lymphocyte count. The same was observed for the neutrophil count (Group C: *p* = 0.90 and A: *p* = 0.80). The neutrophil-to-lymphocyte ratio (NLR) was significantly increased in the DG group compared to C (*p* = 0.04) and A (*p* = 0.04). However, NLR in the DGA group was marginally significant compared to DG (*p* = 0.06) but not different compared to the C (*p* = 1.00) or A (*p* = 1.00) groups. Since the distribution of the data of NLR did not pass the normality tests, the Kruskal–Wallis test was applied, followed by Dunn’s post hoc test.

**Figure 3:**
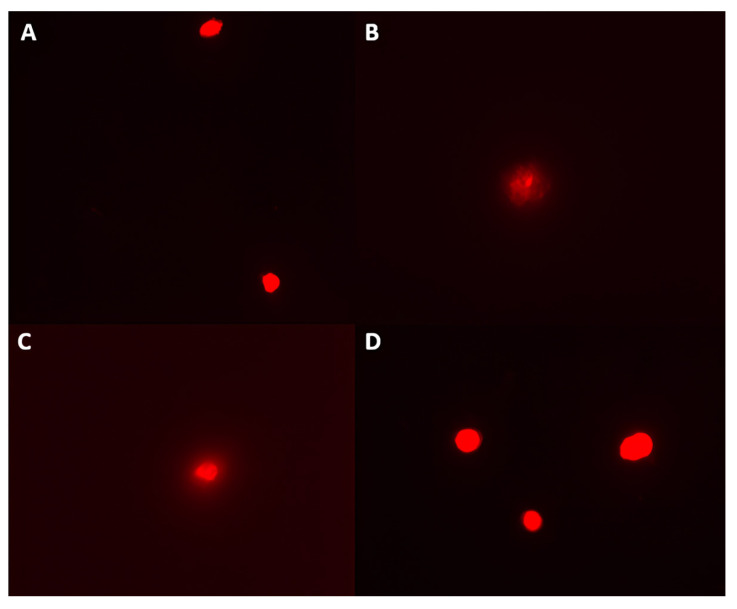
Photomicrographs, taken at 400× magnification, represent leukocytes analyzed using the comet assay in the different experimental groups of the paternal generation (F0): (**A**) Group C showing two leukocytes subjected to horizontal electrophoresis with a low level of damage (Score 0). (**B**) Group DG with a leukocyte showing DNA damage considered intermediate, exhibiting dispersion in the fragmented DNA (Score 2). (**C**) Group DGA with DNA fragmentation considered mild (Score 1). (**D**) Group A with intact DNA structures (Score 0), without evidence of fragment migration (*n* = 5/group).

**Figure 4:**
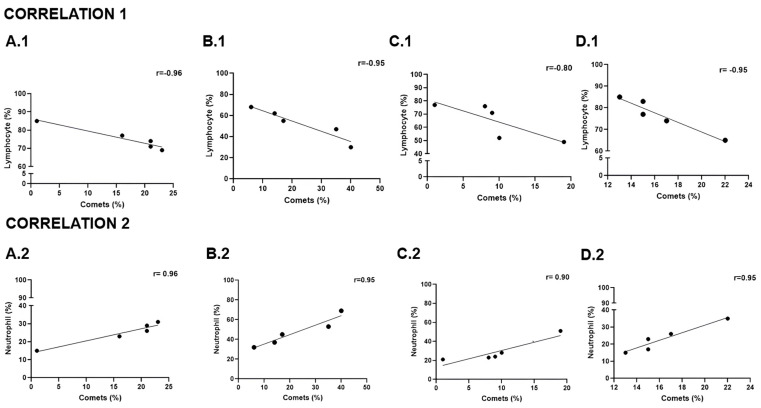
Correlation between lymphocytes, neutrophils and comets (scores 2 + 3) in the paternal generation F0. Correlation 1: Negative correlation between lymphocytes and comets (score 2 + 3) in the paternal generation (F0). Pearson’s correlation was not significant for Group DGA. (**A.1**) Group C (*p* = 0.007); (**B.1**) Group DG (*p* = 0.01); (**C.1**) Group DGA (*p* = 0.09); (**D.1**) Group A (*p* = 0.01). Correlation 2: Positive correlation between neutrophils and comets (score 2 + 3) in the paternal generation (F0). Pearson’s correlation shows significant results in all the studied groups. (**A.2**) Group C (*p* = 0.007); (**B.2**) Group DG (*p* = 0.01); (**C.2**) Group DGA (*p* = 0.03); (**D.2**) Group A (*p* = 0.01).

**Figure 5:**
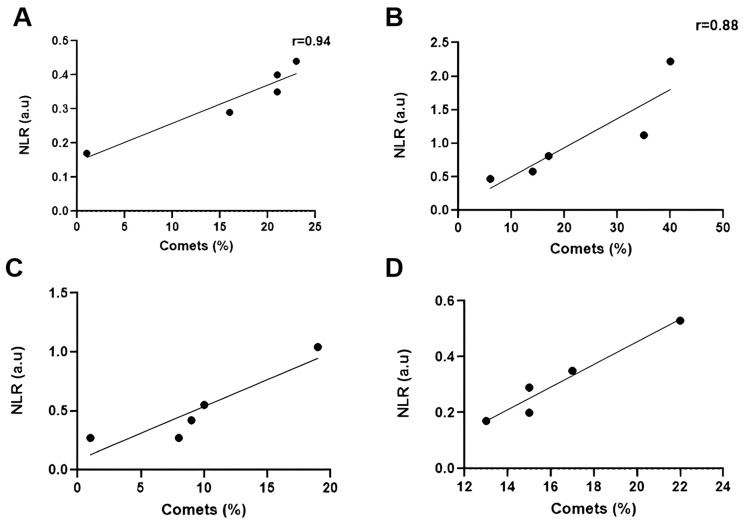
Correlation between NRL and comets (scores 2 + 3) in the paternal generation F0. A positive correlation was detected when comparing the NLR and comets (score 2 + 3) in the paternal generation F0. (**A**) Group C (*p* = 0.01); (**B**) Group DG (*p* = 0.04); (**C**) Group DGA (*p* = 0.02); (**D**) Group A (*p* = 0.005).

In the analysis of the leukocyte DNA integrity, although no significant differences were observed when the groups were compared (Table 2; Figure 3), a positive association was seen with both the lymphocyte/neutrophil counts (Figure 4) or NLR (Figure 5). Interestingly, a significant correlation was also found between sperm motility and NLR, but only for groups treated with açaí (Figure S1).

### 3.3. Vascular Alterations in D-Galactose-Aged Model Were Mitigated by Açaí: Paternal Generation (F0)

**Figure 6:**
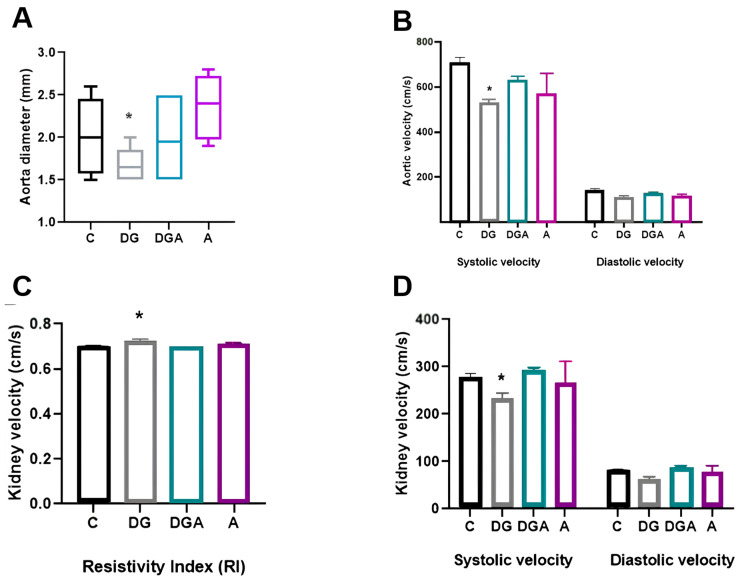
Ultrasound parameters of the paternal generation F0. (**A**) Aorta diameter. * DG < A. Data expressed in median interquartile intervals (*n* = 6/group). (**B**,**D**) Doppler ultrasound measurements for the pulse wave velocity (PWV) in systolic (PSV) and diastolic (PDV) blood flows. * DG < C, DGA and A. (**C**) Renal resistivity index (RI). * DG > C, DGA and A. (**D**) Renal systolic and diastolic pulse velocity. * DG < C and DGA. Data expressed in mean ± standard deviation (*n* = 3/group).

The analysis of the aorta diameter (abdominal region) measured through imaging ultrasound in the DG group was altered only compared to the A group (*p* = 0.03) (C (*p* = 0.48); DGA (*p* = 0.57)), analyzed by Tukey’s post-test (data in Figure 6). The DGA group did not show a significant difference when compared to groups C (*p* = 0.99) and A (*p* = 0.36). Conversely, in aorta systolic velocity (Figure 6B), the DG group was lower compared to groups C, DGA, and A (*p* < 0.0001; *p* = 0.04; *p* = 0.001, respectively). Similarly, a lower systolic velocity was seen in the kidneys (Figure 6D) in the DG group (Group C, *p* = 0.04; DGA, *p* = 0.01), except in Group A (*p* = 0.18). The resistivity index (RI; Figure 6C) was higher in the DG group when compared to the other groups (C: *p* = 0.0003; DGA: *p* = 0.0002; A: *p* = 0.004), but there were no significant differences for the diastolic velocity in the aorta or in the kidneys (Figure 6B and D, respectively). No correlations were found between NLR and aorta diameter or aorta systolic velocity (Figures S2 and S3), as well as between aortic diameter and systolic velocity (Figure S4). On the other hand, a positive correlation was found between renal systolic velocity and NLR only in DG group (Figure S5). Representative echographies of aorta and kidney echostructure are provided in the supplementary material (Figures S6–S9).

### 3.4. First Generation of Males (F1): Paternal Effect on Body Weight, Sperm Morphology Is Partially Mitigated by Açaí

**Figure 7:**
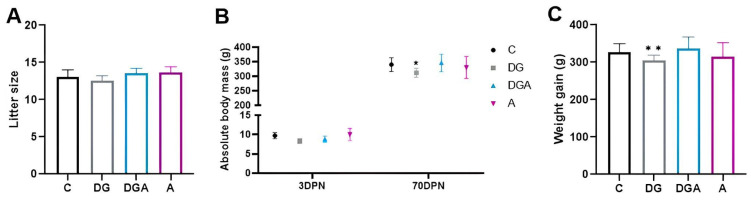
Litter size and body mass of male offspring (F1). (**A**) Litter size. No significant differences found. Mean ± standard deviation (*n* = 3 litter/group). (**B**) The absolute body mass at 3PND did not differ between the groups. * DG < C, DGA e A. (**C**) Weight gain (final versus initial). ** DG < DGA. Data expressed as mean ± SDV. (C: *n* = 13; DG: *n* = 12; DGA: *n* = 12; A: *n* = 15).

The rats treated only with D-galactose and presented to naive females had a slightly smaller litter size (males and females) than the other groups, although no significance was detected (Figure 7A).

The body mass (Figure 7B,C) of the male offspring at 3PND did not present significant differences between the groups (*p* < 0.05). On the other hand, at 70PND, the DG group had reduced body mass compared with the C (*p* = 0.0003), DGA (*p* < 0.0001) and A (*p* = 0.03) groups. Surprisingly, the weight gain (Figure 7C) at 70PND was also decreased when DG was compared with DGA (*p* = 0.01); however, there were no significant differences between DG and the other groups (Group C: *p* = 0.16 and Group A: *p* = 0.72). Furthermore, the DGA group did not differ from any other group for the weight gain (Group C: *p* = 0.74 and Group A: *p* = 0.18). The data were subjected to one-way ANOVA and Tukey’s post-test.

On the other hand, no significant differences were noticed in organ weights of the male offspring (F1) at 70PND (absolute or relative; Table 3). Although the means observed in the kidney and the testis seemed to be marginally significant, the relative weights did not confirm such differences.

**Figure 8:**
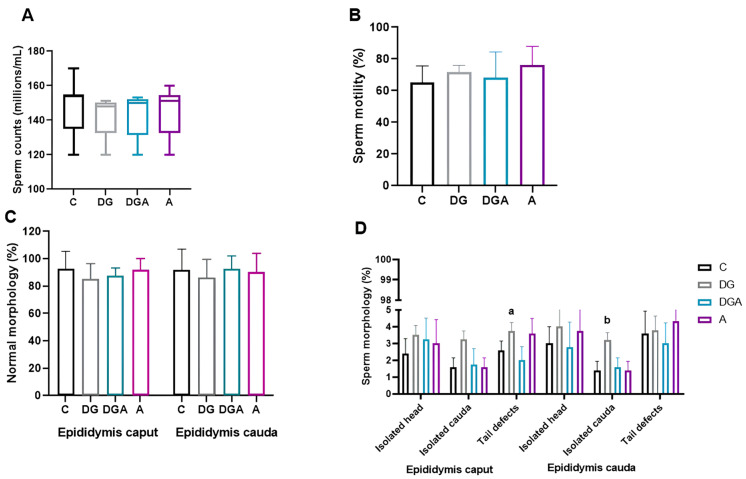
Sperm parameters in the male offspring (F1). (**A**) Sperm concentration distribution (millions/mL). Data expressed as median and interquartile range. (**B**) Motility in samples collected from the epididymis cauda. Data are expressed as mean ± standard deviation. There was no statistical difference in any of the analyzed parameters. Sample sizes in C: *n* = 13; DG: *n* = 12; DGA: *n* = 12; A: *n* = 15. (**C**) Normal sperm morphology. (**D**) Types of morphological alterations (isolated head and cauda, and/or tail defects). a: DG > DGA; b: DG > C and A.

The sperm count, motility and normal morphology (Figure 8) found in F1 did not show significant differences among the groups (one-way ANOVA; *p* = 0.74 for concentration and *p* = 0.29 for motility; Morphology in epididymis head: F = 0.63; *p* = 0.60; in the cauda: F = 0.15; *p* = 0.92). Conversely, differences were observed in the DG group regarding tail defects compared to the DGA group (*p* = 0.04), but not compared to the C (*p* = 0.29) or A (*p* = 0.99) groups. In the epididymis cauda, sperm with isolated tails (b) were increased in the DG group compared to C and A (*p* = 0.03 in both groups) but not compared to DGA (*p* = 0.07).

### 3.5. Paternal Effect on Immune Cells: Leukocyte DNA Vulnerability in the Male Offspring Is Partially Mitigated by Açaí

**Figure 9:**
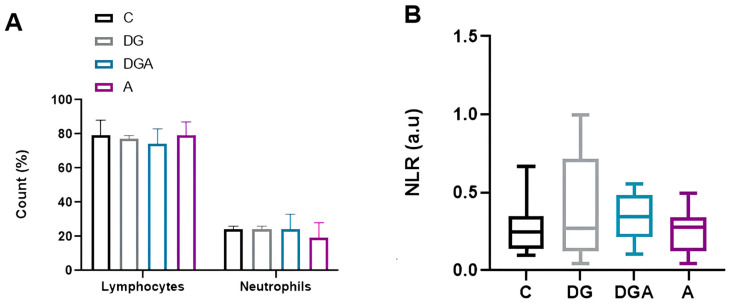
Analysis of leukocyte parameters in males of the first generation (F1). (**A**) Absolute counts of neutrophils and lymphocytes. No significant differences among the experimental groups. (**B**) Neutrophil-to-lymphocyte ratio, also with no significant differences among the experimental groups (*n* = 5/group).

Lymphocyte and neutrophil counts (Figure 9A,B) in the male offspring showed no significant differences between the groups (Figure 9A; two-way ANOVA test; F = 2.42; *p* = 0.07). In Figure 9B, the NLR was also non-significant (Kruskal–Wallis test; *p* = 0.55).

**Figure 10:**
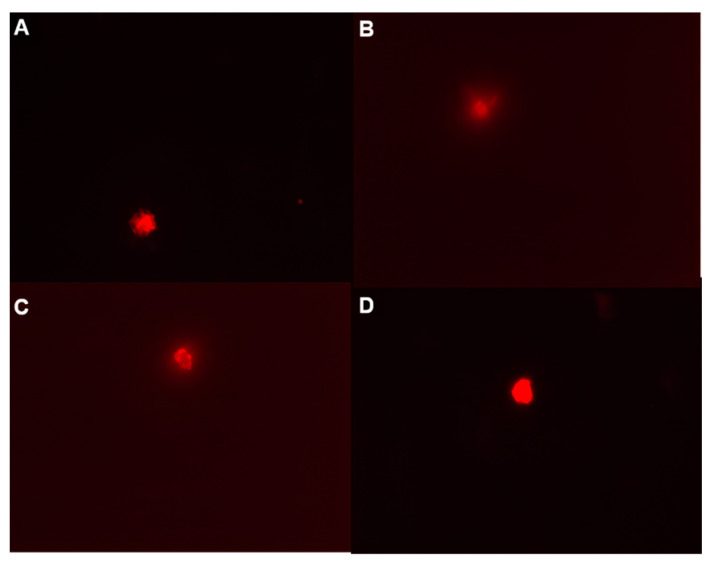
Photomicrographs, taken at 400× magnification, represent leukocytes analyzed using the Comet assay in different experimental groups (F1). (**A**) In Group C, a leukocyte with low damage (Score 1). (**B**) In Group DG, a leukocyte with mild damage (Score 2). (**C**) In Group DGA, a leukocyte with low fragmentation (Score 1). (**D**) In Group A, a leukocyte with no migration of DNA fragments (Score 0).

**Figure 11:**
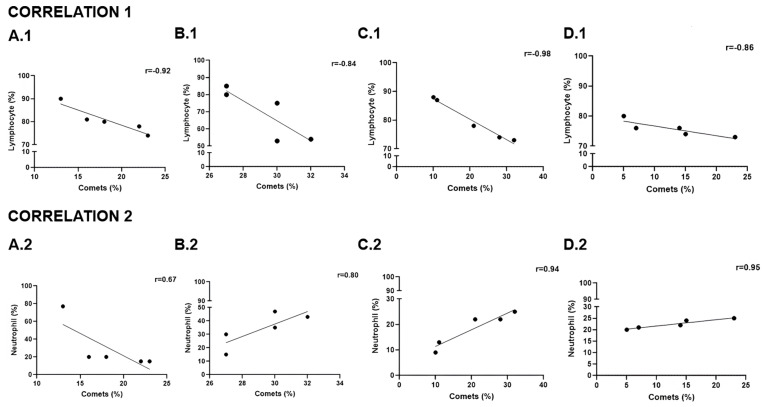
Correlation between lymphocytes and neutrophils in the first generation of males (F1). Correlation 1: Negative correlations between lymphocyte counts and comets (Score 2 + 3) in the first generation of males (F1). Pearson’s test showed significant results in Group C and Group DGA. (**A.1**) Group C; (**B.1**) Group DG; (**C.1**) Group DGA; (**D.1**) Group A. Correlation 2: Positive correlations between neutrophil counts and comets (Score 2 + 3) in the first generation of males (F1). Pearson’s test showed significant results in Group DGA and Group A. (**A.2**) Group C; (**B.2**) Group DG; (**C.2**) Group DGA; (**D.2**) Group A.

**Figure 12:**
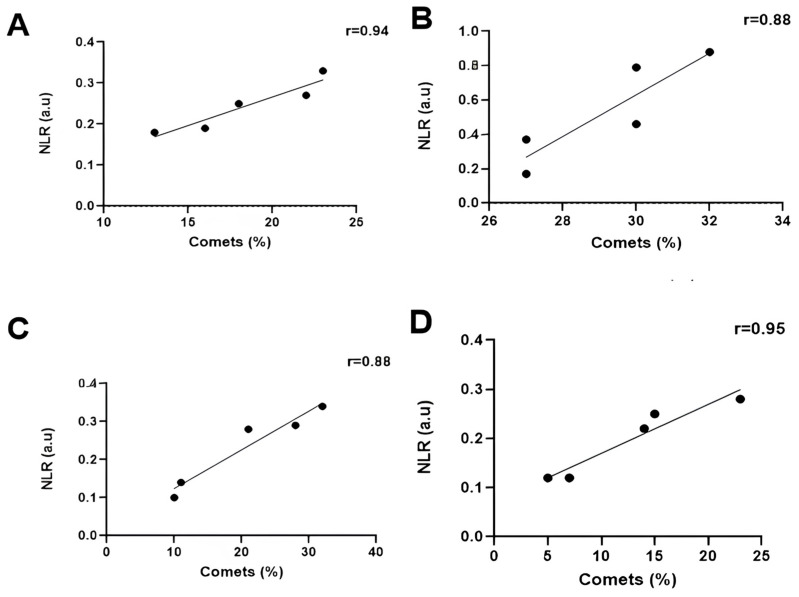
Positive correlation between NLR and comets (score 2 + 3) in the first generation of males (F1). Pearson’s test showed significant results in all groups. (**A**) Group C; (**B**) Group DG; (**C**) Group DGA; (**D**) Group A.

On the other hand, the number of leukocytes containing severe DNA fragmentation (score 3; Table 4; Figure 10) was significantly increased in the DG group when compared to C and A (*p* = 0.01), while in the DGA group, no significant differences were found (*p* = 0.11). The DGA group showed no alterations compared to Group C (*p* = 0.59) and A (*p* = 0.68). In the analysis of the damaged cells scored 2 and 3 together (cells with moderate or severe fragmentation, termed as “comets”; Table 4; Figure 10), the number of leukocytes in the DG group was also higher than in the Group C (*p* = 0.04) and Group A (*p* = 0.004), while the DGA group showed no significant differences (*p* = 0.11). Conversely, the DGA group showed no alterations compared to Group C (*p* = 0.96) and Group A (*p* = 0.33). Interestingly, negative correlations were seen in Group DGA for lymphocyte counts and comets (score 2 + 3) in the first generation of males (Figure 11); similarly, a positive correlation between neutrophil and comets (score 2 + 3) was identified in the Group C and Group DGA (Figure 11): Group C (*p* = 0.02); Group DG (*p* = 0.07); Group DGA (*p* = 0.001); Group A (*p* = 0.06). Moreover, Figure 12 shows that the NRL was positively correlated with comets in all the groups: Group C (*p* = 0.01); Group DG (*p* = 0.04); Group DGA (*p* = 0.007); Group A (*p* = 0.01). These results indicate a higher vulnerability of the immune system cells transmitted by the D-galactose-aging model, pointing to a successful mimicking of APA. Conversely, açaí’s nutraceutical effect was partially seen in leukocyte integrity maintenance.

### 3.6. No Significant Vascular Alterations in the Male Offspring (F1)

**Figure 13:**
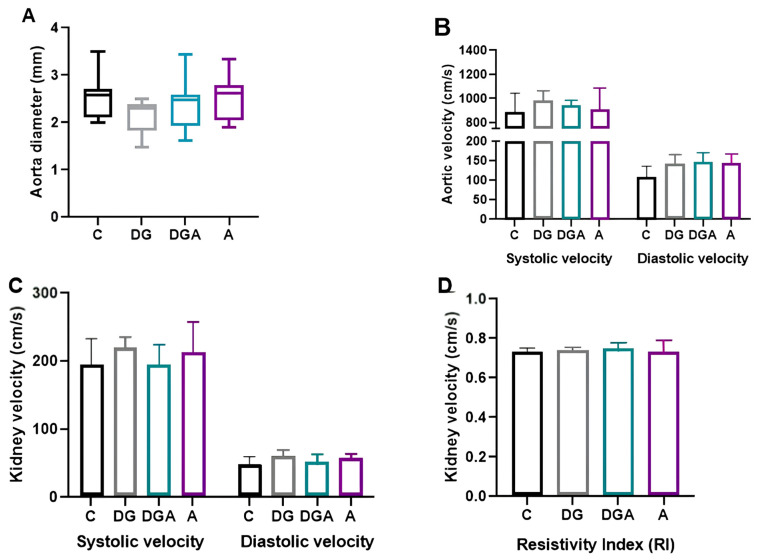
Ultrasound parameters of first-generation males (F1). (**A**) Aorta diameter (*n* = 10/group) (**B**) Doppler ultrasound measurements for the pulse wave velocity (PWV) in systolic (PSV) and diastolic (PDV) blood flows. (**C**) Pulse wave velocity (PWV) in systolic (PSV) and diastolic (PDV) renal blood flows. (**D**) Renal resistivity index (RI). No significant differences were found. Means ± standard deviation (*n* = 8/group).

**Figure 14:**
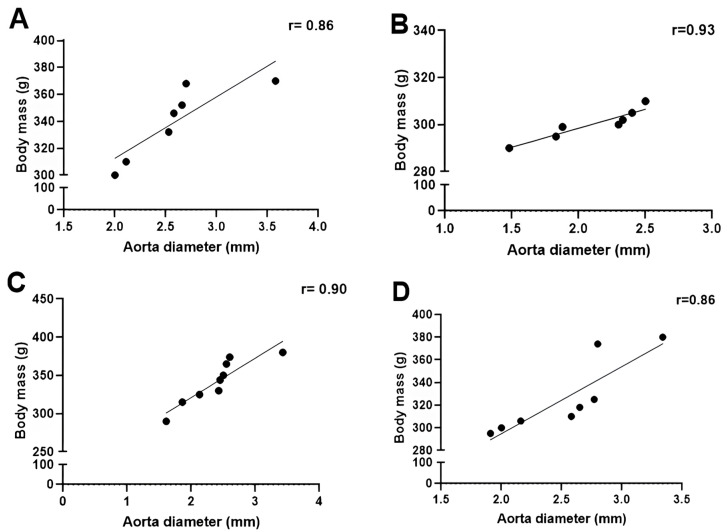
Positive correlations between body mass (70DPN) and aortic diameter in the first generation of males (F1). Group C (**A**), Group DG (**B**), Group DGA (**C**) and Group A (**D**). Pearson’s test showed significant results in all groups (*n* = 8/group).

The abdominal aorta diameter did not differ among the groups (ANOVA one-way; F = 1.41; *p* = 0.26). Pulse wave velocity (PWV) in systolic (PSV) and diastolic (PDV) blood flows, neither in the aorta nor in the kidney, was altered (Figure 13). On the other hand, a positive correlation was found between body mass and aortic diameter (Figure 14), which was statistically significant in all the groups: Group C (*p* = 0.01); Group DG (*p* = 0.002); Group DGA (*p* = 0.001); Group A (*p* = 0.005).

## 4. Discussion

Advances in modern medicine have enabled an unprecedented increase in human longevity, which may not be accompanied by a proportional expansion of functional health. Likewise, extended longevity is not directly associated with a safe reproductive healthspan, and from the perspective of intergenerational biological transmission, some alterations may represent increased risk to the general health and the quality of life of the descendants [26,58].

The effects of 200 mg/kg of D-galactose on the male reproductive system in rodents have been described previously, starting from a 6-week treatment [59]. More recently, a study showed that 4-week treatment with a slightly lower dose of D-galactose (150 mg/kg b.w.) increases senescence-associated β-galactosidase activity, causing REDOX imbalance and apoptosis in cardiac tissue in rats [60]. In our protocol, we were able to show that a short treatment with D-galactose induces systemic aging alterations, mimicking a window in male reproduction comparable to natural aging and APA-related outcomes seen in clinical practice. Here, we present alterations that suggest initial (or more vulnerable) signs of systemic alterations, with implications for the offspring. The effects of D-galactose were detected in parameters known to be more prone to oxidative stress—sperm with low motility and normal morphology, increased NRL, and blood flow alterations, even without causing infertility, but successfully increasing intergenerational health risks, thus mimicking an APA-like effect in the D-galactose-induced aging model.

A growing number of studies have been using this model to investigate the effects of aging on the reproductive system, such as increased peroxidation of membrane lipids, damage to the antioxidant system in germ cells, and the morphology and motility of spermatozoa [61,62,63], a panorama not necessarily seen in the APA scenario. However, to the best of our knowledge, this is the first study reporting the consequences of D-galactose age induction on paternal inheritance, thus contributing to amplifying the application of this experimental model.

In a previous study, our group found that the 4-week protocol of aging induction produces increased numbers of apoptotic germ cells and a threshold effect on sperm DNA damage, along with incipient systemic effects, making it challenging to trace the whole health decay (data not published). Herein, the results corroborate that the rats are already under physiological challenge, augmenting the risk of paternal adverse contribution, probably through harming the male germline production and maturation.

The neutrophil and lymphocyte counts have been largely used in routine investigations, but NLR is increasingly considered a significant biomarker and has gained importance in the context of various diseases, including those associated with cellular senescence [36]. The NLR can be indicative of systemic inflammaging status [64], which is linked to the cellular senescence process [64,65]. Peripheral leukocyte DNA fragmentation is a marker of cellular damage, often caused by oxidative stress, environmental toxins, or disease states, which can be measured to assess overall genotoxicity and health risks [66]. It is commonly evaluated using the comet assay (single-cell gel electrophoresis) in studies regarding cancer risk, smoking habits, or exposure to hazardous substances. Sperm with low motility, also known as asthenozoospermia, is another marker of oxidative stress and mirrors systemic inflammation statuses, such as those seen in metabolic diseases [34].

A clear negative effect of the treatment with D-galactose in paternal inheritance was found in the protocol proposed here, which can be related to the increase in inflammatory status [67,68], as suggested in our results. Although the lyophilized açaí promoted broad protection against aging acceleration in the treated males, seen not only in neutrophil modulation but also in aorta diameter, systolic velocity, and renal resistivity index, it did not prevent the alterations seen in the leukocyte fragmentation in male offspring. On the other hand, the fact that for the leukocyte DNA integrity seen in the offspring, the DGA group was not significantly different from the C and A groups indicates a slight positive biological effect of açaí, which could result in a more resilient immune system in the offspring, an aspect not completely addressed yet.

Additionally, to corroborate this observation, the significant association between leukocyte quantitative parameters (lymphocyte, neutrophils and NRL) and the higher levels of DNA fragmentation suggests an important hematological vulnerability in the male offspring caused by paternal inheritance. Through the accumulation of advanced glycation end-products, D-galactose aging in rodents creates a challenging environment for the immune system, increasing cytokine production [67] and decreasing lymphocyte mitogenesis [68], comparable to natural aging [54,55].

High oxidative stress and inflammation are events directly associated with methylation modifications of sperm, reduced sperm function, and infertility [69]. Indeed, as men age, loss of methylation in certain CpG sites associated with inflammatory responses may increase the inflammatory state of semen [70,71]. Whether sperm underwent changes in their epigenetic marks and if such modifications are capable of transgenerational transmission are aspects not focused on here and warrant future investigation.

Here, we confirmed that the epididymis is primarily susceptible to systemic inflammation and immune reactions [71], which potentially threatens the acquisition of small non-coding RNA cargo and membrane modification for sperm competence [72,73,74]. Because sperm have no active transcription following spermiogenesis, the RNA load carried by the sperm ensures the regulation of zygotic gene expression [75], which is indispensable for preimplantation development [73,74]. Indeed, the epigenetic marks carried by the sperm are critical mediators between environment and intergenerational health [76].

Paternal aging effects on the offspring can be very complex, being evident as early as the first lineage of differentiation during preimplantation development [76,77,78], which may be associated with low birth weight and prematurity [8,10]. Similar vulnerabilities were seen in the offspring of the D-galactose-aging model fathers, which had reduced weight gain and changes in sperm and leukocyte parameters. Moreover, the strong positive correlation between both the increase in NLR and in leukocyte DNA fragmentation indicated a similar trend towards a favorable inheritance provided by açaí [79]. However, the parameters studied here have limited ability to confirm the extent of protection or to predict susceptibility to developing diseases.

On the other hand, a linear association between advanced paternal age and hematological disturbances [80] or lymphoblastic leukemia has been reported [17,81,82]. Even after the exclusion of hereditary genetic syndromes, an increase in the relative risk of amyloid lymphoblastic leukemia (ALL) was observed, estimated at a 1.13 rate for each five-year increment in paternal age [79]. In this matter, it may become a growing public health challenge for future generations, given the high prevalence of this neoplasm in developed countries [17].

Systemic decline in the aging process can be silent and not necessarily accompanied by remarkable health symptoms, such as infertility, but some key alterations can be detected, as shown here. REDOX signaling and pro-inflammatory cytokine production were not carried out with this protocol, but the systemic changes seen here were probably driven by mechanisms involving inflammaging and oxidative stress.

## 5. Conclusions

The results of this study indicate that the protocol of aging induction with D-galactose, with voluntary consumption and using melon as a vehicle for a period of thirty days, induced detectable systemic implications, evidenced in reproductive, immunological and vascular parameters. Açaí showed high nutraceutical power, mitigating most of the alterations in all the aspects studied in the male’s health (paternal generation). Although the protocol did not result in infertility in the rats, an intergenerational effect was observed in the male offspring, indicating an increased vulnerability to the immune system of the first generation.

In our study, intergenerational transmission was not completely attenuated by preconception use of açaí, but biological indications of protection and modest signs of modulation of the alterations stand out; however, these results must be more broadly investigated. Adjustments in the protocol—e.g., in the dose of açaí—might also be needed to achieve enhanced health outcomes for descendants.

## Figures and Tables

**Table 1:** Body mass and organ weights (absolute and relative) in the paternal generation (F0) for the groups C, DG, DGA and A.

GROUPS F0					
Body Mass and Organ Weights (g)	C (*n* = 6)	DG (*n* = 6)	DGA (*n* = 6)	A (*n* = 6)	*p*
Body Mass	399 ± 25.89	395 ± 24.68	389 ± 22.08	391 ± 7.62	0.87
Kidney	1.68 ± 0.17	1.73 ± 0.16	1.76 ± 0.20	1.73 ± 0.28	0.90
Kidney (RW)	0.42 ± 0.05	0.43 ± 0.04	0.45 ± 0.05	0.44 ± 0.06	0.78
Liver	12.90 ± 1.35	13.00 ± 0.82	13.66 ± 2.19	13.25 ± 1.44	0.93
Liver (RW)	3.24 ± 0.21	3.30 ± 0.19	3.51 ± 0.56	3.39 ± 0.04	0.72
Epididymis	0.64 ± 0.05	0.75 ± 0.05 *	0.65 ± 0.05	0.67 ± 0.05	0.03 *
Epididymis (RW)	0.16 ± 0.01	0.19 ± 0.01 **	0.16 ± 0.01	0.17 ± 0.01	0.02 **
Testis	1.85 ± 0.19	1.90 ± 0.13	1.93 ± 0.08	1.93 ± 0.12	0.78
Testis (RW)	0.46 ± 0.04	0.48 ± 0.02	0.49 ± 0.04	0.49 ± 0.04	0.34

Statistical Analysis: one-way ANOVA. Significant when *p* < 0.05. Mean ± SDV; RW: relative weight. * DG > C and DGA; ** DG > C and DGA.

**Table 2:** Classification of leukocyte profiles subjected to the Comet assay in F0.

	GROUPS F0				
	C (*n* = 5)	DG (*n* = 5)	DGA (*n* = 5)	A (*n* = 5)	*p*
Score 0	6.40 ± 13.22	7.40 ± 6.98	16.80 ± 17.69	6.40 ± 10.69	0.74
Score 1	78.00 ± 5.43	58.60 ± 28.05	70.40 ± 13.24	76.60 ± 9.96	0.44
Score 2	15.00 ± 8.66	17.40 ± 10.01	7.60 ± 6.58	15.40 ± 3.43	0.22
Score 3	1.40 ± 0.89	5.00 ± 5.83	1.80 ± 1.78	0.60 ± 0.54	0.20
Comets	16.40 ± 8.99	22.50 ± 14.47	9.40 ± 6.43	16.40 ± 3.44	0.24

Statistical Analysis: One-way ANOVA (mean ± standard deviation).

**Table 3:** Absolute and relative weights of organs collected from the male offspring (F1) fathered by the D-galactose-aged young rats treated or not with açaí. Values expressed as mean ± SDV.

GROUPS F1 (Male Offspring)					
Organ Weight (g)	C (*n* = 13)	DG (*n* = 12)	DGA (*n* = 12)	A (*n* = 15)	*p*
Kidney	1.68 ± 0.31	1.49 ± 0.10	1.67 ± 0.38	1.65 ± 0.28	0.06
Kidney (RW)	0.50 ± 0.08	0.48 ± 0.03	0.48 ± 0.10	0.50 ± 0.10	0.69
Liver	14.00 ± 2.97	13.67 ± 1.31	13.83 ± 2.20	15.00 ± 3.93	0.69
Liver (RW)	4.21 ± 0.95	4.39 ± 0.42	4.02 ± 0.71	4.57 ± 1.22	0.40
Epididymis	0.55 ± 0.12	0.56 ± 0.16	0.58 ± 0.13	0.58 ± 0.09	0.87
Epididymis (WR)	0.16 ± 0.03	1.18 ± 0.05	0.17 ± 0.04	1.18 ± 0.04	0.68
Testis	1.91 ± 0.18	1.80 ± 0.10	1.80 ± 0.46	1.98 ± 0.22	0.06
Testis (RW)	0.57 ± 0.05	0.58 ± 0.02	0.52 ± 0.13	0.61 ± 0.80	0.33

Statistical Analysis: One-way ANOVA. RW = relative weight.

**Table 4:** Classification of leukocyte DNA integrity subjected to the comet assay in the first generation of males (F1). Mean ± standard deviation (*n* = 5).

GROUPS F1					
	C	DG	DGA	A	*p*
Score 0	2.40 ± 0.54	1.20 ± 0.44	3.80 ± 6.26	11.80 ± 23.59	0.52
Score 1	84.80 ± 3.11	74.00 ± 8.80	81.20 ± 5.54	77.80 ± 19.62	0.48
Score 2	16.80 ± 3.42	19.60 ± 2.07	15.40 ± 5.98	10.80 ± 7.25	0.08
Score 3	1.60 ± 0.89	11.40 ± 6.65	5.00 ± 5.05	2.00 ± 0.70	0.01 *
Comets (2 + 3)	18.40 ± 4.15	31.00 ± 4.95	20.40 ± 9.86	12.80 ± 7.15	0.01 **

Statistical Analysis: One-way ANOVA. * DG > C and A (*p* = 0.01). ** DG > C and A (*p* = 0.04 and 0.004, respectively).

## Data Availability

The raw data supporting the conclusions of this article will be made available by the authors upon request.

## Supplementary Materials

The following supporting information can be downloaded at: https://www.mdpi.com/article/10.3390/biology15131086/s1, Figure S1: Positive correlation using Pearson’s test between sperm motility and NLR in the paternal generation (F0); Figure S2. Pearson correlation between NLR and aorta diameter in the paternal generation (F0); Figure S3. Pearson’s test correlation between aortic systolic velocity and NLR in the paternal generation (F0); Figure S4. Pearson’s test correlations in aortic systolic velocity and aortic diameter in the paternal generation (F0); Figure S5. Pearson’s test correlation between renal systolic velocity and NLR in the paternal generation (F0); Figure S6. Echography of aortic echostructure in the paternal generation F0; Figure S7. Echography of the right kidney echostructure in the paternal generation (F0); Figure S8. Echographies of aortic echostructure in males of the first generation (F1); Figure S9. Echography of the right kidney echostructure in males of the first generation (F1).
