# Supplementary material for: Sperm, Neutrophil and Vascular Alterations in Advanced Paternal Age Model and the Nutraceutical Effect of Açaí to Mitigate Health Vulnerability in the Male Offspring
Source: Biology (Basel). 2026 Jul 6;15(13):1086. doi: 10.3390/biology15131086 (PMC13359979; doi:10.3390/biology15131086)
Supplement: Supplementary file 1 [file biology-15-01086-s001.zip › biology-4304017-supplementary.pdf]

biology

MDPI

Supplementary Materials

Complementary analyses of the parameters observed in the paternal generation (F0):

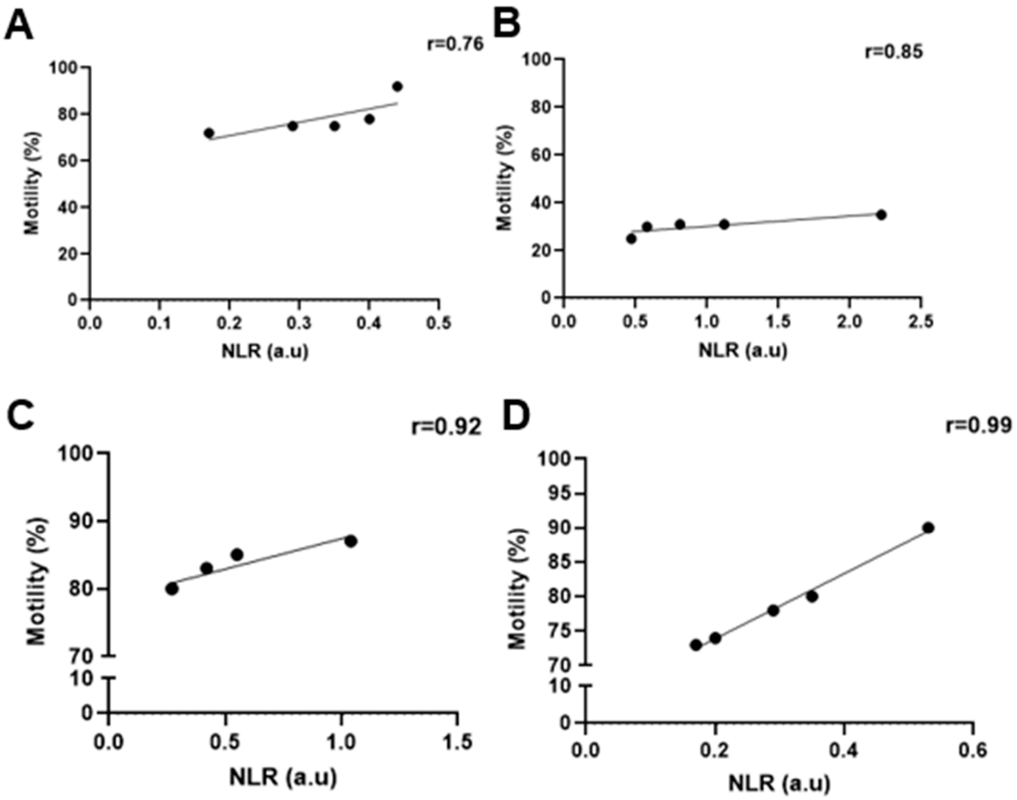

Figure S1. Positive correlation using Pearson's test between sperm motility and NLR in the paternal generation (F0). Pearson's correlation was significant in the groups DGA and A. **22A**: Group C (p = *0.12*); **22B:** Group DG (p = *0.06*); **22C:** Group DGA (p = *0.02*); **22D:** Group A (p = *0.0003*).

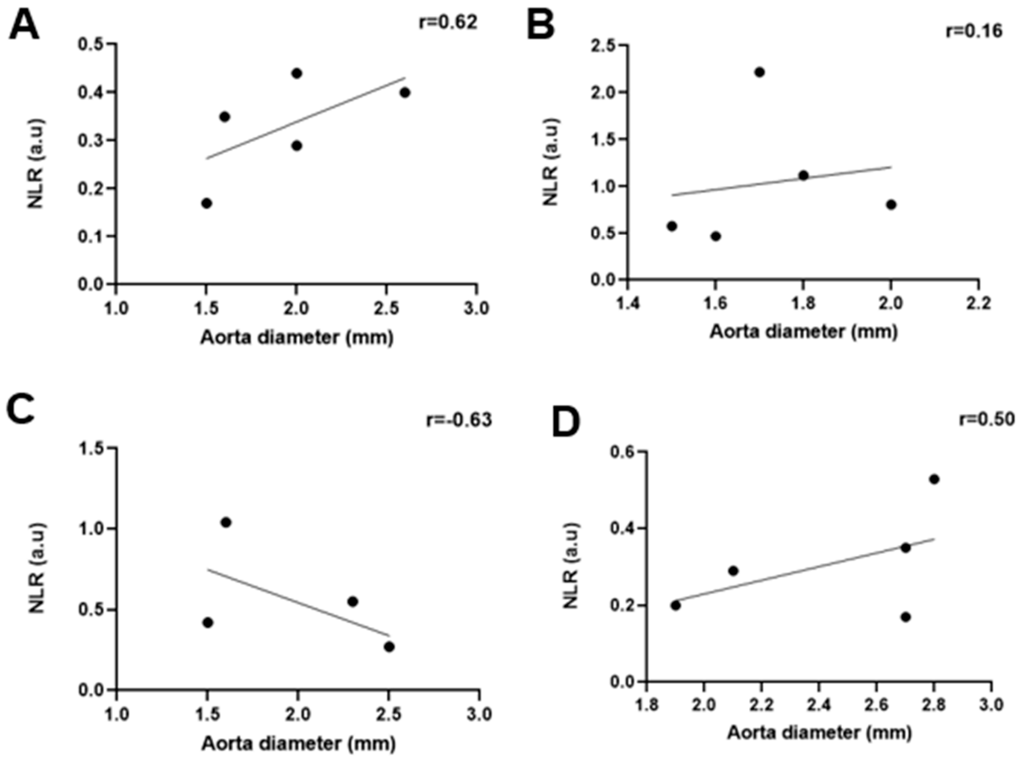

Figure S2. Pearson correlation between NLR and aorta diameter in the paternal generation (F0). No significant differences were found. **(A)** Group C *(p = 0.26)*; **(B)** Group DG *(p = 0.79)*; **(C)** Group DGA *(p = 0.25)*; **(D)** Group A (p = *0.38*).

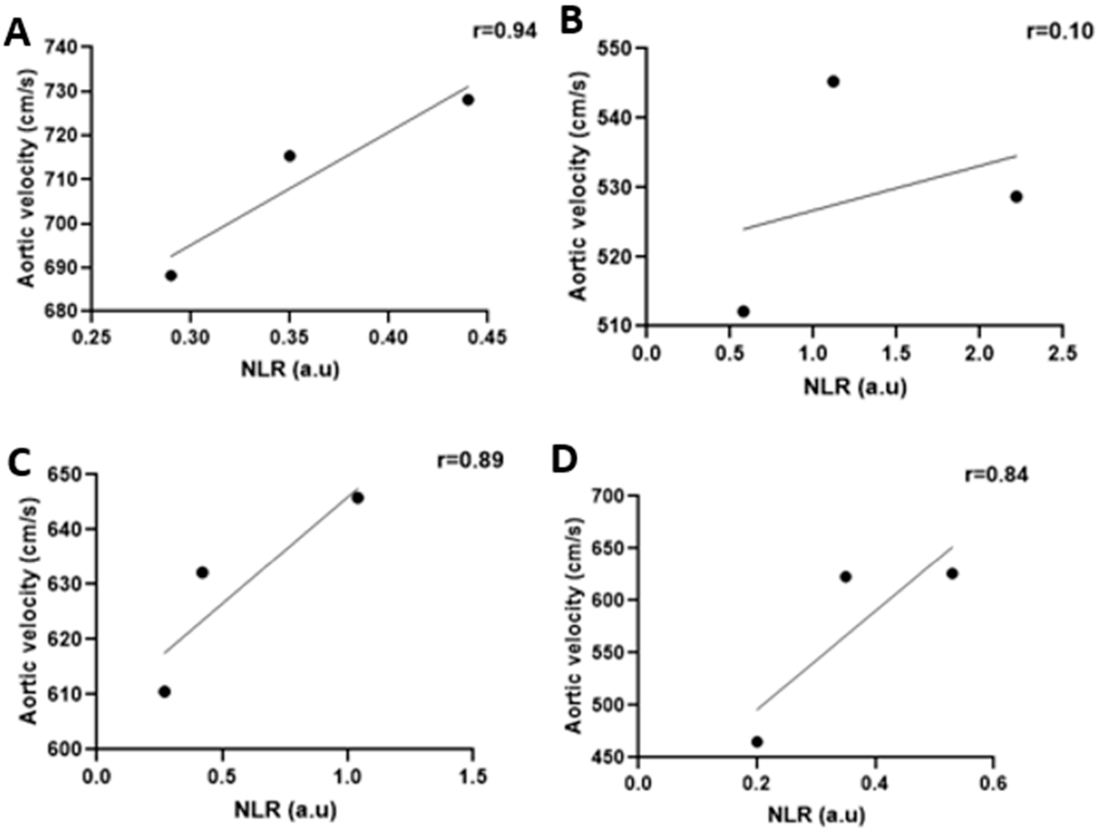

Figure S3. Pearson's test correlation between aortic systolic velocity and NLR in the paternal generation (F0). No significant differences were found. **(A)** Group C (*p = 0.20*); **(B)** Group DG (*p = 0.79*); **(C)** Group DGA (*p= 0.25)*; **(D)** Group A (*p= 0.38*).

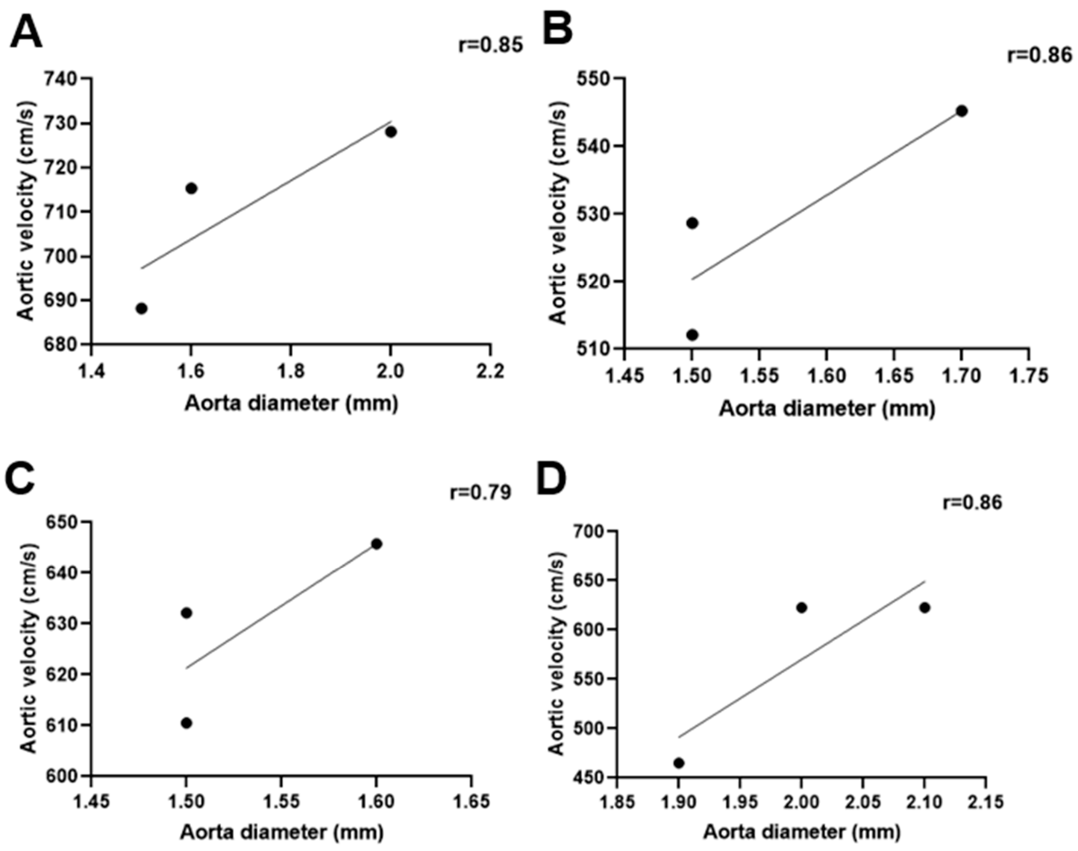

Figure S4. Pearson's test correlations in aortic systolic velocity and aortic diameter in the paternal generation (F0). No significant differences were found. (A) Group C ($p = 0.34$); (B) Group DG ($p = 0.33$); (C) Group DGA ($p = 0.41$); (D) Group A ($p = 0.33$).

https://doi.org/10.3390/biology15131086

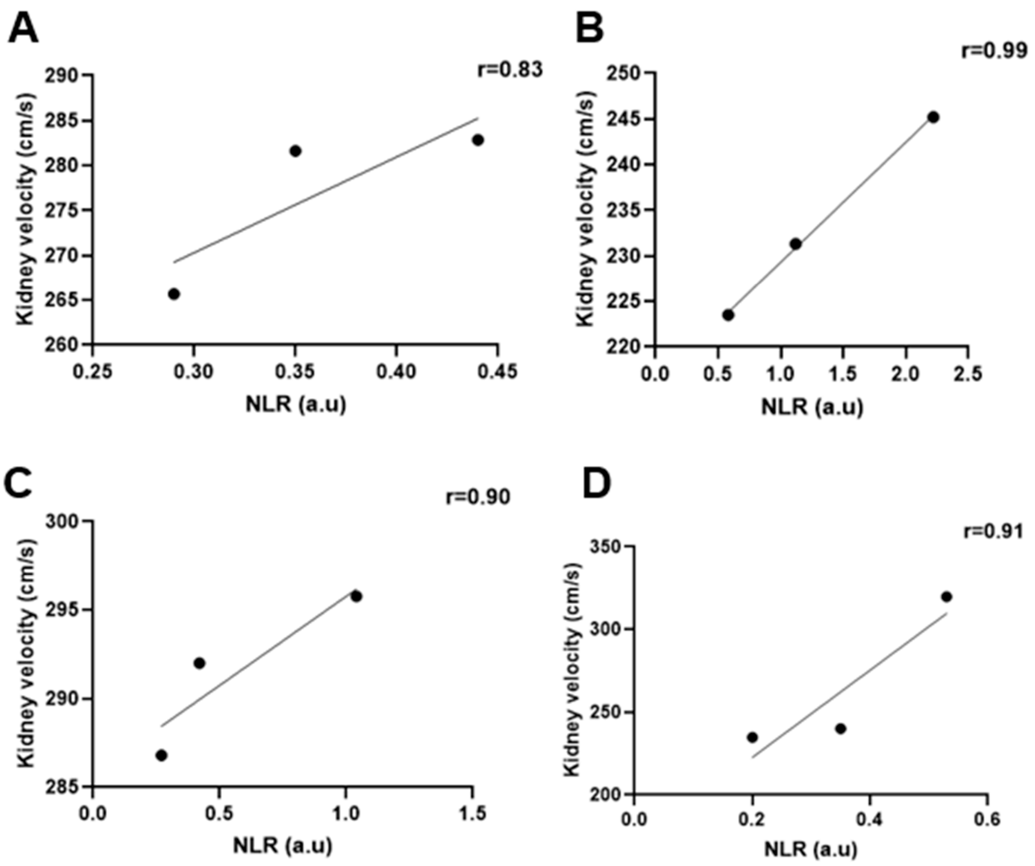

Figure S5. Pearson's test correlation between renal systolic velocity and NLR in the paternal generation (F0). Significant differences were found only in Group DG. **(A)** Group C (*p* = *0.36*); **(B)** Group DG (*p* = *0.02*); **(C)** Group DGA (*p* = *0.27*); **(D)** Group A (*p* = *0.83*).

2. Echographies observed in the male first generation (F1):

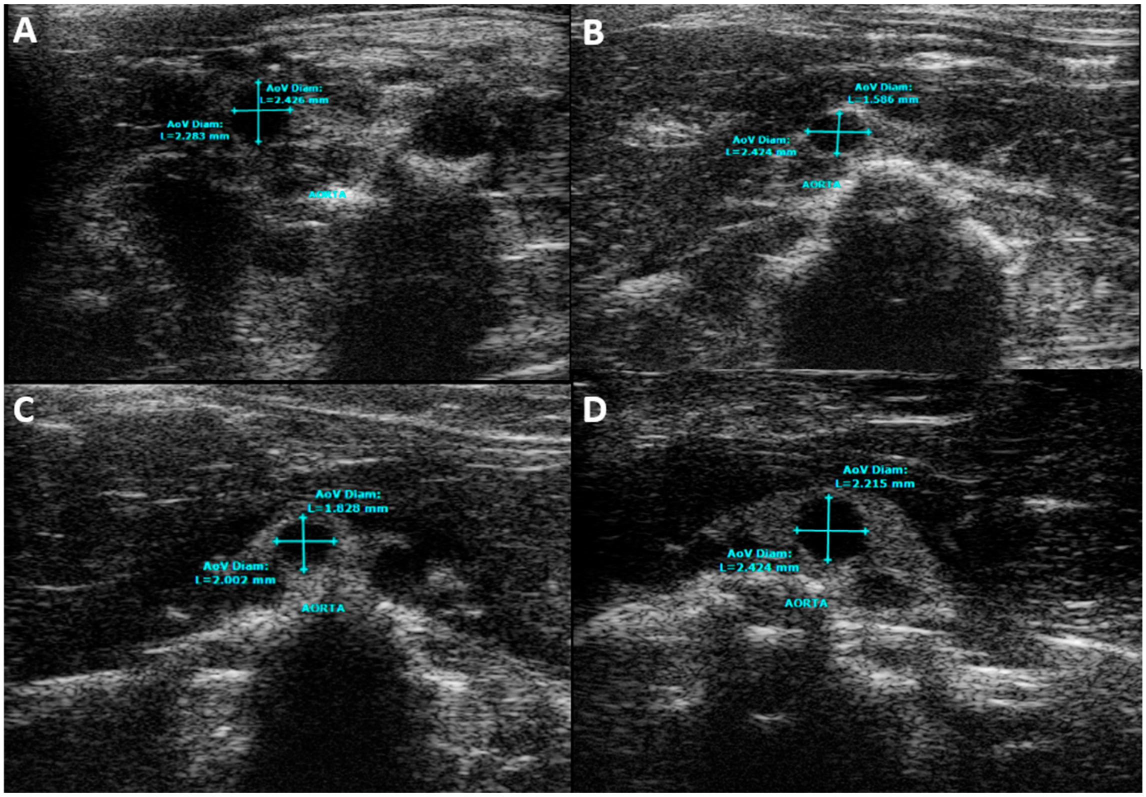

Figure S6. Echography of aortic echostructure in the paternal generation F0. **(A)** Group C; **(B)** Group DG; **(C)** Group DGA; **(D)** Group A.

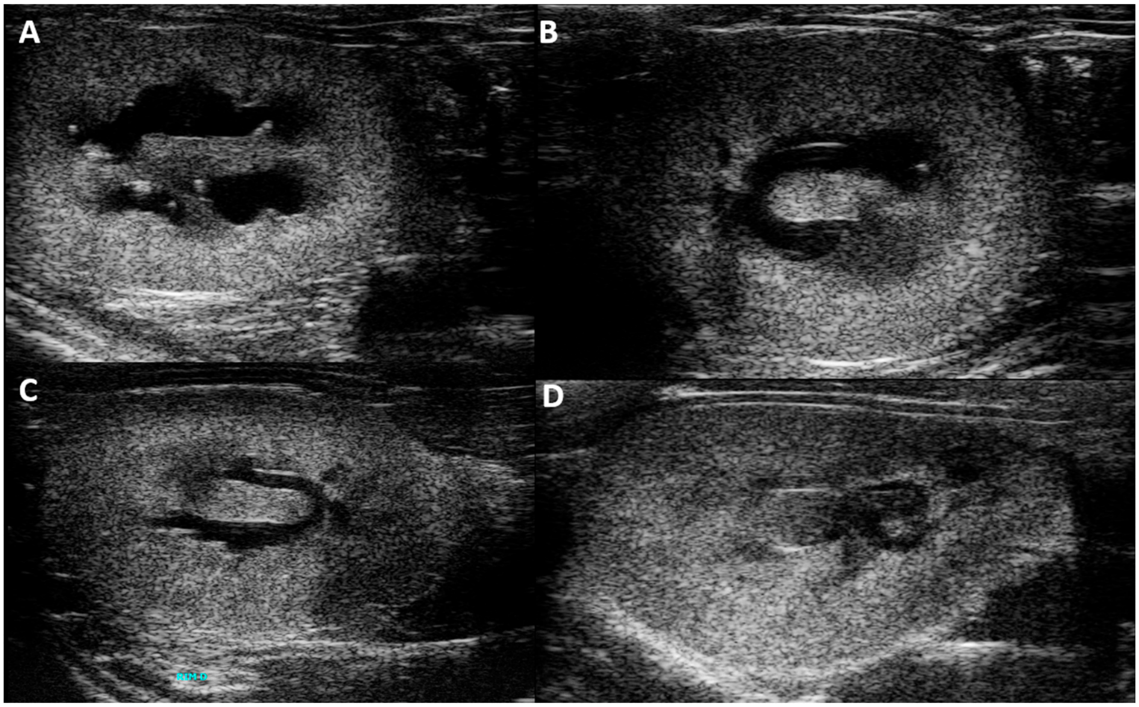

Figure S7. Echography of the right kidney echostructure in the paternal generation (F0). **(A)** Group C; **(B)** Group DG; **(C)** Group DGA; **(D)** Group A.

https://doi.org/10.3390/biology15131086

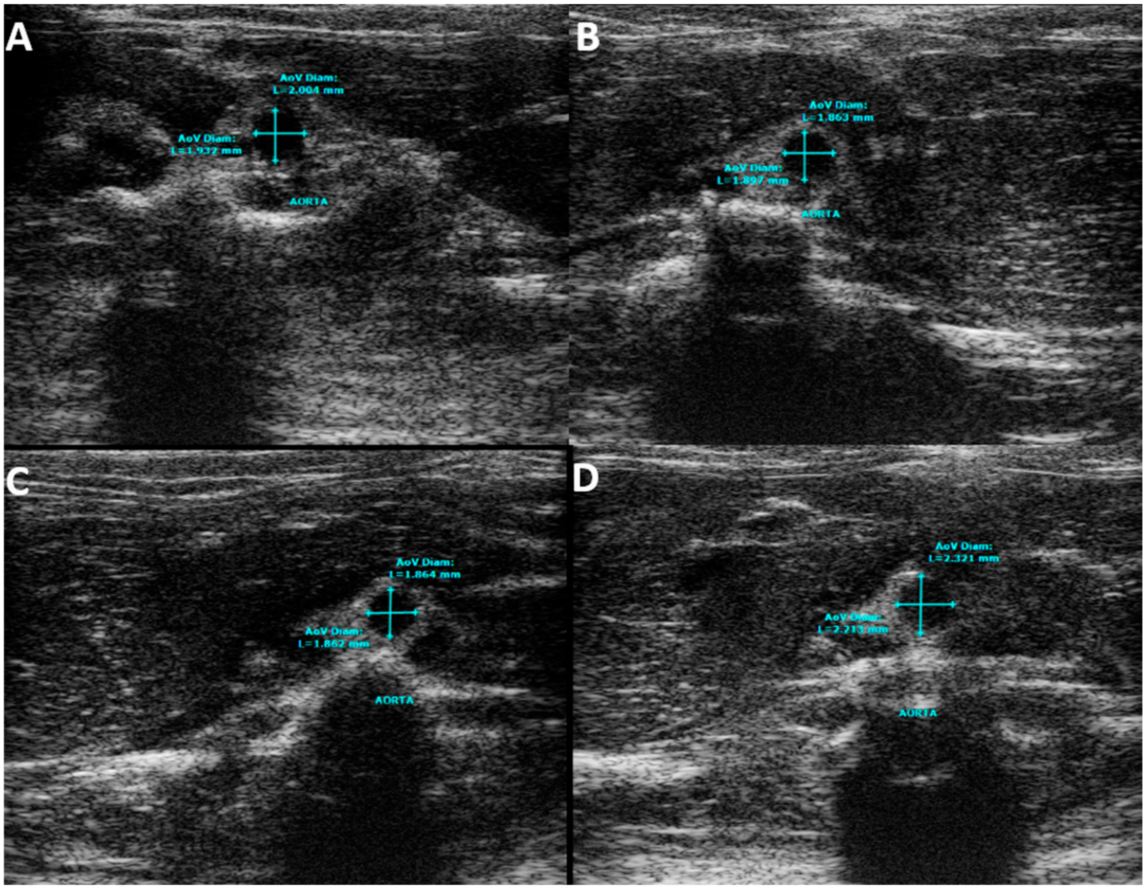

Figure S8. Echographies of aortic echostructure in males of the first generation (F1). **(A)** Group C; **(B)** Group DG; **(C)** Group DGA; **(D)** Group A.

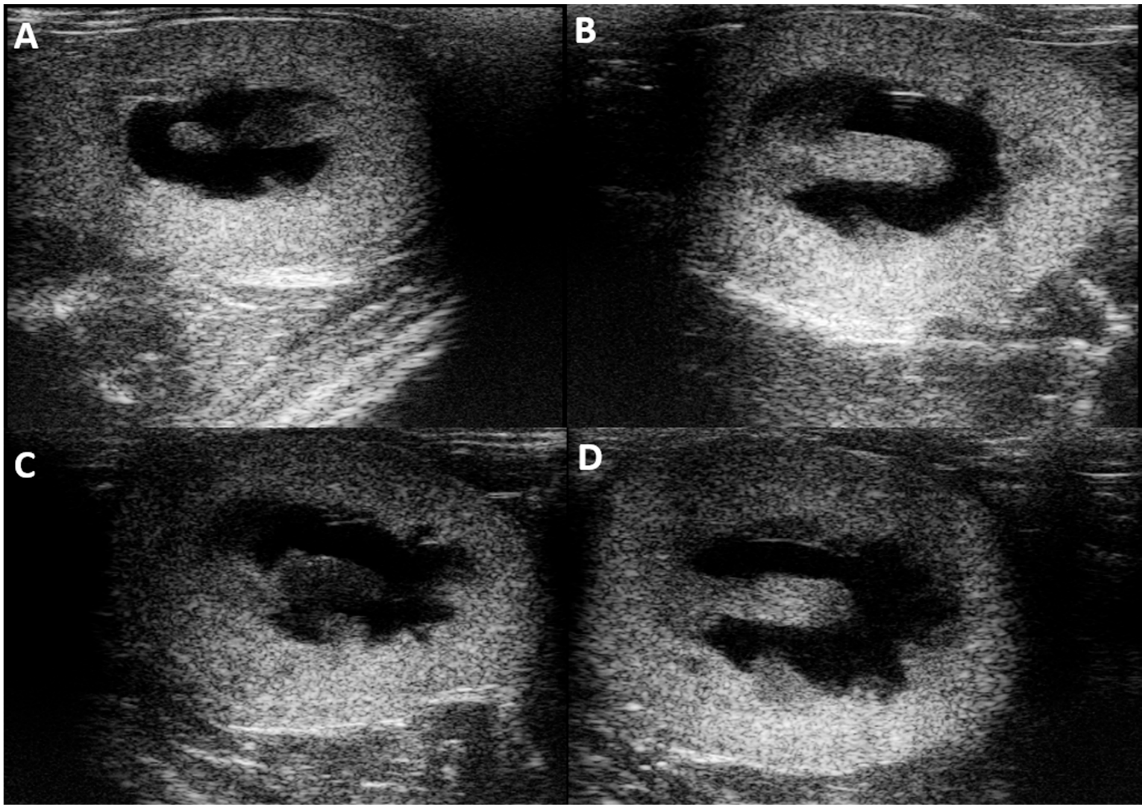

Figure S9. Echography of the right kidney echostructure in males of the first generation (F1). **(A)** Group C; **(B)** Group DG; **(C)** Group DGA; **(D)** Group A.

Disclaimer/Publisher's Note: The statements, opinions and data contained in all publications are solely those of the individual author(s) and contributor(s) and not of MDPI and/or the editor(s). MDPI and/or the editor(s) disclaim responsibility for any injury to people or property resulting from any ideas, methods, instructions or products referred to in the content.

https://doi.org/10.3390/biology15131086
